# Audit of Annual Blood Tests for Patients on Antipsychotic Medications in the Recovery Team

**DOI:** 10.1192/bjo.2024.532

**Published:** 2024-08-01

**Authors:** Omolayo Apantaku, Mohamed Yaasir Mohamudbucus, Bettahalasoor Somashekar, Ashok Kumar, Daniela Borges

**Affiliations:** Coventry & Warwickshire Partnership NHS Trust, Coventry, United Kingdom

## Abstract

**Aims:**

All patients who are prescribed antipsychotic medications require annual blood tests which must include Full Blood Count (FBC), Urea and Electrolytes (U&E), eGFR, Lipids (Cholesterol & Triglycerides), Liver Function Test (LFT), HbA1c/Plasma glucose. Some patients also require prolactin blood test depending on their prescribed antipsychotic medication.

NICE and Maudsley guidelines recommend an annual check of the blood tests mentioned above.

This audit ascertained compliance in terms of annual blood test monitoring for patients who take antipsychotic medications and provided recommendations to improve where necessary.

**Methods:**

Half of the caseload from two General Adult psychiatry Community Mental Health Teams (CMHT) were recruited from a sample population of 228 patients. Odd number randomisation was applied to select our sample (e.g., 1, 3, 5…). Sample size was of 114 patients, 8 of whom were not prescribed antipsychotic medications and excluded. Hence, 106 patients were identified as representative for inclusion in this audit.

Retrospective data collection was from clinical entries, clinic letters and blood test results.

Data obtained from these patients was collated and analysed using MS Excel spreadsheet.

**Results:**

The audit revealed that compliance was suboptimal for all required blood tests (Compliance 80% or above is recommended). The kidney function test of Urea & Electrolytes was the closest to recommended standards and best performance overall (77%), eGFR, was subpar at 60%. 74% of patients had the glucose monitoring tests (Plasma glucose/HbA1c) done while Full Blood Count and Liver Function Test were both completed in 76 patients **(~72%)**. The worst performing category was Prolactin monitoring, of which only 9 of patients who required this had it done, recording a mere 31%.

Majority (66%) of the blood tests were done at General Practice (GP) surgeries, 25% by Mental Health Services, while the rest were contributed to by Accident & Emergency and Acute Hospital visits.

**Conclusion:**

Following completion of this audit, recommendations were made to advise existing antipsychotic blood monitoring services (GP surgeries and private clinic affiliated with the Trust) of the recommended blood parameters for monitoring, and the need to update current systems. Also, Liaison with service managers and service leads to set up a dedicated physical health clinic for this purpose. The latter has been particularly successful as the Trust is now in the process of recruitment for the new physical health clinic team. A re-audit is planned in the near future.

